# Social media recruitment enhances participant diversity in dermatology clinical trial: findings from the SAFA trial

**DOI:** 10.1186/s13063-025-08994-5

**Published:** 2025-08-27

**Authors:** Cherish Boxall, Susanne Renz, Beth Stuart, Ingrid Muller, Irene Soulsby, Jacqueline Nuttall, Karen Thomas, Kim S. Thomas, Tracey H. Sach, Megan Lawrence, Matthew J. Ridd, Nick Francis, Paul Little, Zina Eminton, Gareth Griffiths, Alison M. Layton, Alan Saji, Charlotte Cluff, Miriam Santer

**Affiliations:** 1 Southampton Clinical Trials Unit, University of Southampton, Southampton, UK; 2 Primary Care Research Centre, School of Primary Care, Population Sciences and Medical Education, Faculty of Medicine, University of Southampton, Southampton, UK; 3 Public Contributor, Southampton, UK; 4 Centre of Evidence Based Dermatology, Lifespan and Population Health, School of Medicine, University of Nottingham, Nottingham, UK; 5 Centre for Applied Excellence in Skin & Allergy Research, University of Bristol, Bristol, UK; 6 Hull York Medical School, University of York, York, UK; 7 Faculty of Medicine, University of Southampton, Southampton, UK

**Keywords:** Social media, Recruitment, Retention dermatology, Acne, Online recruitment, Randomised controlled trial

## Abstract

Recruitment and retention of participants remain critical challenges in clinical trials, often requiring innovative approaches to ensure sufficient enrolment and sustained engagement. Social media advertising offers the potential to reach target populations quickly by leveraging demographic, geographic and interest-based targeting. This mixed-methods observational study evaluates participant experiences and the effectiveness of various recruitment routes within a trial of a treatment for acne. Demographic variables, including age, ethnicity, acne severity and acne duration, were stratified primary care, secondary care, community and social media recruitment routes and 12 participant interviews were analysed using reflexive thematic analysis. Social media recruitment accounted for over half of participants (53.9%). It was particularly effective in recruiting individuals with higher acne severity (IGA ≥ 3; 57.5% of its recruits, *n* = 127), longer duration of disease (> 5 years history of acne; 60.2% of its recruits, *n* = 133) and from ethnic minority groups (9.0% of its recruits, *n* = 20), the latter being notably higher than the proportion recruited via primary care (1.5% of its recruits, *n* = 1). Slight variations in retention by recruitment route were observed, with social media (85%) and primary care (84%) achieving the highest retention rate at the 12-week follow-up. All routes lost between 25 and 32% of participants by the 24-week follow-up, signifying the importance of implementing effective retention strategies to keep participants engaged. Overall, participants found targeted social media advertisements to be an acceptable and convenient recruitment approach; initial signals of trust were provided by high-quality graphics and recognisable NHS and university logos, which coupled with responsive trial staff, were suggested to provide a seamless enrolment experience. This study demonstrates that social media recruitment can be an effective and acceptable component of a multi-route strategy for clinical trial enrolment.

## Background

The recruitment of participants is one of the largest and most persistent challenges in clinical trials [1–3] and one of the most important elements linked to a trial’s success.

Social media, defined as ‘websites that let users make profiles and use these profiles to connect and interact with other individuals’ [4], has been increasingly experimented with as a digital recruitment tool for various disease areas. Social media advertising offers the possibility to reach a target population based on demographics, geographic location and interests in a shorter amount of time [5, 6], thus differentiating this digital tool from conventional recruitment approaches (e.g. targeted mailings, direct recruitment, posters/leaflets).

Despite the increasing use of social media advertisement to enhance clinical trial recruitment in practice [7], there are limited comprehensive evaluations of this approach to assess its overall impact on the number of people reached, recruited and retained, as well as the characteristics of these individuals. An understanding of the attitudes of different participant populations towards social media recruitment is also lacking [8], which could uncover influencers to engagement. Combined with real-time web usage analysis and trial recruitment metrics, evaluation data could enhance the effectiveness of future social media recruitment campaigns.

People living with skin conditions have previously been shown to access skin or cosmetic-related information on different social media platforms [9], suggesting a large, engaged and accessible potential population for recruitment to research. In this paper, we describe the impact of social media on an acne randomised controlled trial (RCT) called SAFA.

## Methods

We aim to (1) compare the conversion rates between individuals reached via social media advertisements and those invited through participant identification centres (PICs) for enrolment in the SAFA trial, (2) determine the characteristics of participants recruited through these different routes and (3) compare the proportion of participants retained per recruitment route. SAFA participants’ attitude towards social media recruitment was also explored through a nested qualitative sub-study.

### Study overview

The SAFA study, described in detail elsewhere [10–12], was a phase 3 multicentre double-blind RCT with two parallel groups (1:1) evaluating the effectiveness and cost-effectiveness of spironolactone compared to placebo for the treatment of acne (in combination with standard topical therapy): the primary outcome was at 12 weeks post-baseline, with the treatment period lasting until 24 weeks post-baseline followed by an unblinded follow-up period for up to 6 months. Results showed that more participants in the spironolactone arm reported acne improvement than in the placebo group: no significant difference was reported at week 12 (72% v 68%, odds ratio 1.16 (95% confidence interval 0.70 to 1.91)), and a significant difference was seen at week 24 (82% v 63%, 2.72 (1.50 to 4.93)).

Participants eligible for the SAFA trial were women aged 18 years or over with facial acne persisting for at least 6 months, severe enough to warrant oral antibiotic treatment and with an investigator’s global assessment of acne (IGA) of at least 2 (mild or worse).

Ethical approval for the SAFA trial was given by the Wales Research Ethics Committee (REC) 3 in January 2019 (18/WA/0420) and Health Research Authority (IRAS246637). The trial was registered prospectively in ISRCTN (ISRCTN12892056) and EudraCT (2018–003630-33).

### Recruitment strategy

Referred to as recruitment routes, potential participants were advertised to through primary care, secondary care, community and social media advertising. Trial consent took place in person in secondary care dermatology clinics. Between June 2019 and August 2021, with an enforced COVID-19 pause from 23 March 2020 to 11 June 2020, 10 dermatology centres were recruiting participants for the study.

### Primary and secondary care sites

Potential participants were identified through targeted mailings by general practices (GPs) local to the recruiting study centres, opportunistically in secondary care dermatology outpatient clinics and screening of new referral letters by all study centres. Mailings by GP surgeries excluded patients who had opted out of being contacted about research.

### Community advertising

**Fig. 1:**
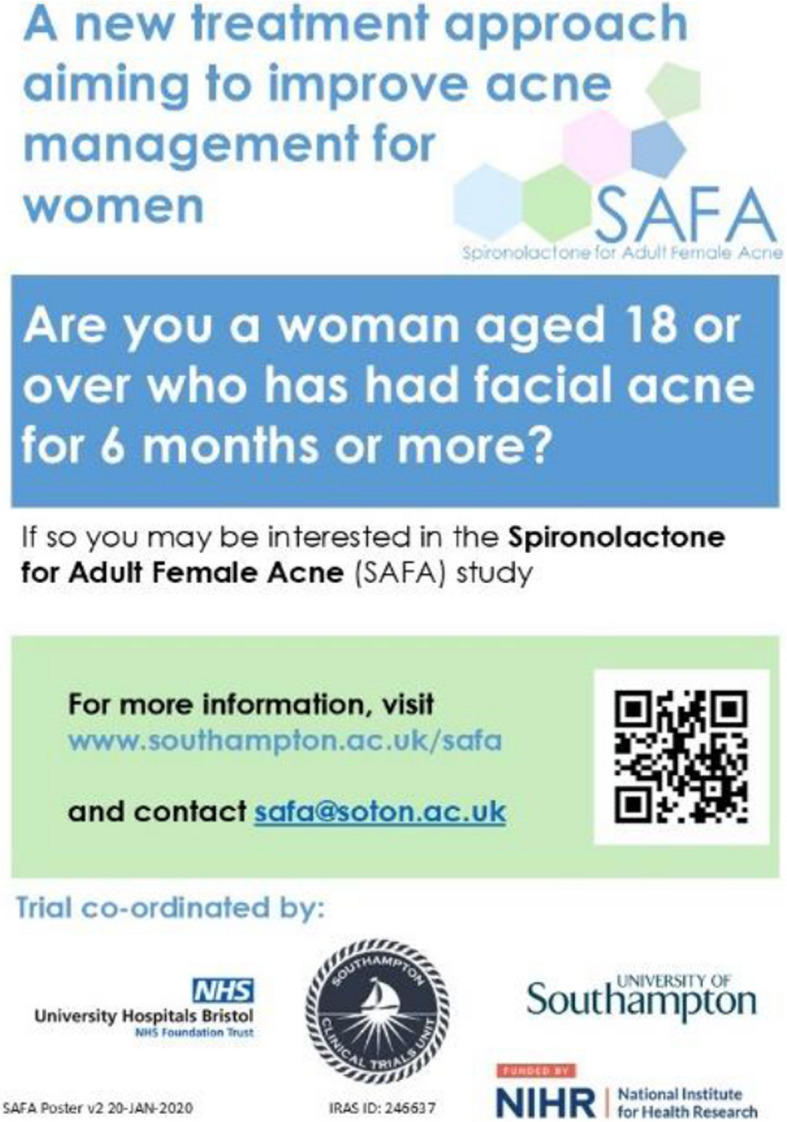
Trial advertisement poster

Trial posters (Fig. 1) were on display in hospitals, pharmacies and universities local to recruiting sites. The adverts directed potential participants to the study website for information about the study (participant information sheet) and contact details to Clinical Trial Unit staff that triaged interested people to the closest recruiting site.

### Social media advertising

Paid social media advertisements were used between December 2019 and August 2021, initially coordinated by a digital marketing company for 2.5 months before being run in-house by Southampton CTU (Clinical Trials Unit) for 14 months. Targeted advertisements (Fig. 1) ran on Facebook, audience networks (a network of websites which are Facebook partners and allow ads to run from Facebook on their website) and Instagram.

**Fig. 2:**
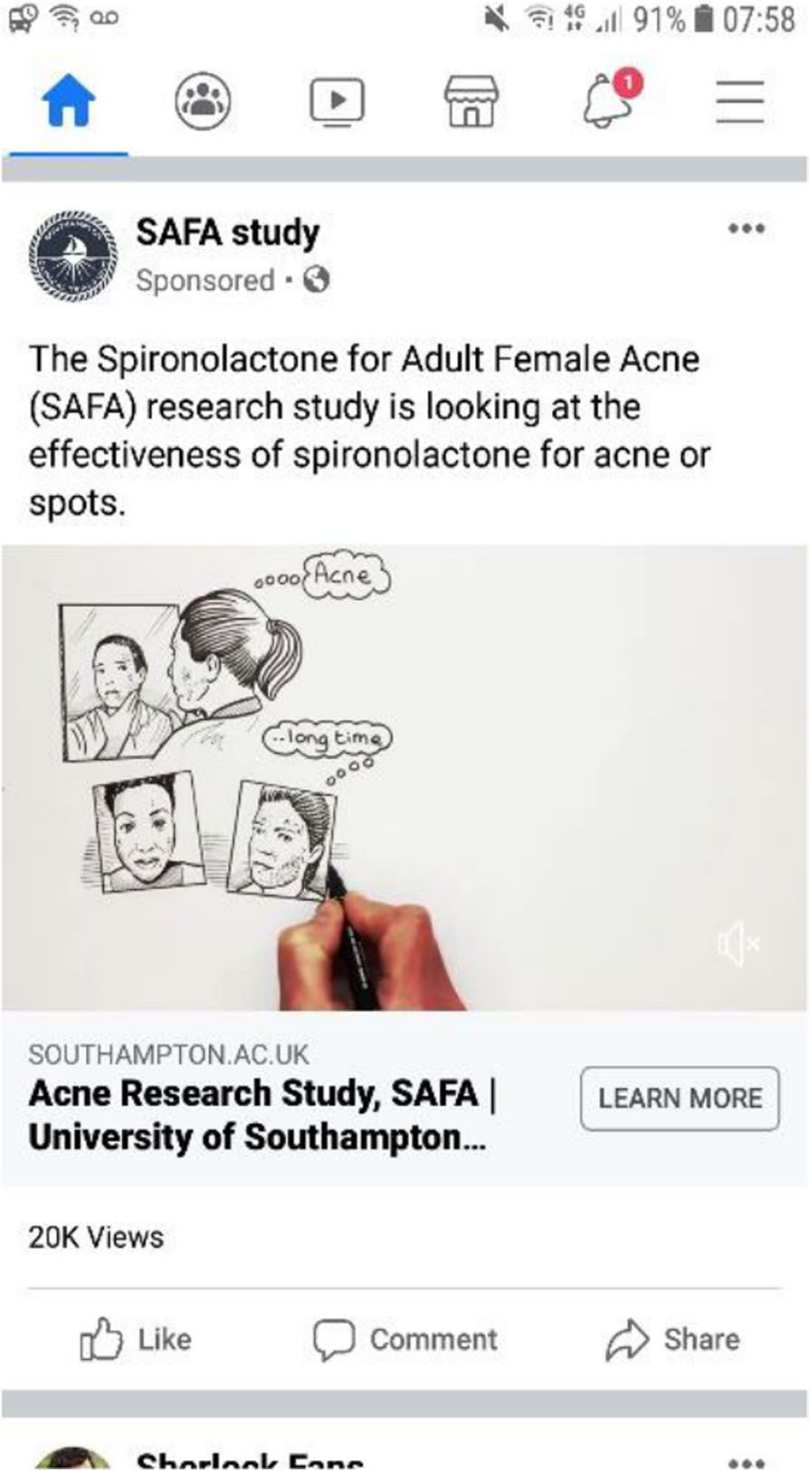
Image of SAFA social media video advert

Adverts comprising 15-s videos explaining the study were targeted to specific audiences on Facebook, using parameters including age (18 +), gender (female) and geographic location (approximately 40 km radius around the respective recruitment centre). These adverts targeted users with interests in acne and/or acne treatments. Viewers could access more study information by clicking through to the study-specific website and expressing their interest in participating by emailing a generic study email account managed by Southampton CTU staff. Southampton CTU staff then directed potential participants to their nearest recruitment centre. Ad campaigns were typically run over weekends (Friday to Monday midday) when they attracted more traffic (Fig. 2).

### Data collection

#### Quantitative data

Facebook Ads Manager (an online tool which helps users manage paid advert campaigns) was used to collect measures of interest: we used (i) age, ethnicity, (ii) impressions: number of times the advert was on-screen, (iii) reach: number of people who saw the advert at least once and (iv) unique link clicks: number of people who performed a link click to study-specific website. Trial data collected from recruiting sites included acne severity and duration.

#### Qualitative data

Participants enrolled in the SAFA trial were invited to take part in an optional qualitative interview with no monetary incentive. Participants were invited to take part opportunistically (based on their willingness and ability) by research staff at sites or by unblinding letters at 24 weeks. The total number of invited participants by research sites is unknown because they were not requested to keep a log, and 174 were invited through an unblinding letter sent 28 weeks after their baseline visit. Trial participants who were interested in being interviewed were asked to email the SAFA qualitative study mailbox accessed by the researchers conducting interviews (CB, AS, CC), who then responded to them via email. Thirteen participants expressed an interest in interview. One participant became unavailable to interview due to personal commitments. Twelve SAFA trial participants took part in a remote semi-structured interview to share their perspectives and thoughts on taking part in the SAFA trial [13]; this paper reports specifically on the topic of social media advertisement.

### Data analysis

#### Quantitative data

Descriptive statistics were used to report and compare the source of recruitment and characteristics of people recruited by primary care, secondary care, community or social media advertising.

#### Qualitative data

Three members of the research team (two medical students and one research fellow), independent from the SAFA trial, conducted the interviews (CB, AS, CC) between October 2021 and February 2022. All identifiable data were anonymised, and quotes were labelled with pseudonyms at the write-up. Interviews had a median duration of 28 min (range 19–39). Data from the interviews were analysed using reflexive thematic analysis [14, 15] to develop themes around participants’ experiences of recruitment to, participation during and healthcare experiences following the SAFA study. CB led the analysis through familiarisation of the data, and generation of codes and themes which were continually refined through the analysis process. CB met regularly with MM, IM and AS to challenge assumptions and highlight the most important patterns in the data.

All interviews were audio recorded then transcribed verbatim, error-checked and data were handled using NVivo10. Data specific to study participants’ perspectives and experiences of SAFA social media advertising are reported here.

## Results

In total, 51,891 people clicked on the social media advert which took them to the study website displaying an information sheet and next steps to express an interest. Five hundred forty people (1% of people reached) continued onto telephone screening by the study team and 221 (0.43% of enrolled vs reached) women enrolled on the study.

In comparison, 2058 women received an invitation pack from their GP practice (*N* = 32), enclosing the same information as the study website. One hundred eight (5.3%) women contacted the Clinical Trials Unit and were screened for eligibility and 65 (3.16% of enrolled vs reached) women enrolled on the study. Despite the low conversion rate between reached and enrolled from social media, overall, this route of advertisement was active for the shortest period and was accountable for 53.9% of enrolled participants (Table 1).

**Table 1:** Overall number of people reached, screened, not enrolled and enrolled in each recruitment route

Recruitment route	Reached (*n*)	Screened (*n*, % of total screened)	Not enrolled (*n*, % of total not enrolled)	Enrolled (*n*, % of total enrolled)
Primary care	2058^¥^	108 (8.5%)	62 (7.3%)	65 (15.9%)
Secondary care	Unknown	425 (33.5%)	323 (37.7%)	87 (21.2%)
Community	Unknown	143 (11.3%)	99 (11.7%)	37 (9%)
Social media	51, 891^&^	540 (42.6%)	319 (37.3%)	221 (53.9%)
Unknown	N/A	51 (6%)	51 (6%)	N/A
Total	53, 949	1267	857	410

^¥^Number of patients who received invitation pack through targeted mailing; the number of patients reached or recruited through opportunistic recruitment is unknown

^&^Clicks to the study website via advert link

The recruitment data suggests that leveraging multiple recruitment channels, including both traditional healthcare routes and digital/community-based approaches, may help recruit a diverse study population in terms of both acne characteristics and demographic representation (Table 2).

**Table 2:** Participant characteristics and clinical characteristics by route of recruitment

	Overall (*n*, %)	Primary care (*n*, %)	Secondary care (*n*, %)	Community (*n*, %)	Social media (*n*, %)
Total participants	410	65	87	37	221
Age					
Mean age (SD)	29.1 (7.2)	28.7 (6.4)	28.9 (6.9)	27.9 (5.3)	29.2 (7.2)
Age range	18–59	18–57	18–59	18–51	18–54
Ethnicity					
White	328 (80.0)	60 (92.3)	71 (81.6)	27 (72.9)	170 (76.9)
Ethnic minority	28 (6.8)	1 (1.5)	4 (4.6)	3 (8.1)	20 (9.0)
Asian or Asian British	9 (2.2)	1 (1.5)	1 (1.1)	1 (2.7)	6 (2.7)
African, Black British or Caribbean	6 (1.5)	0 (0.0)	0 (0.0)	1 (2.7)	5 (2.3)
Mixed	9 (2.2)	0 (0.0)	1 (1.1)	1 (2.7)	7 (3.2)
Another group	4 (1.0)	0 (0.0)	2 (2.3)	0 (0.0)	2 (0.9)
Prefer not to say	33 (8.0)	3 (4.6)	5 (5.7)	3 (8.1)	22 (10.0)
Unknown	21 (5.1)	1 (1.5)	7 (8.0)	4 (10.8)	9 (4.1)
History of acne					
6 months to 2 years	104 (25.4)	18 (27.7)	28 (32.2)	15 (40.5)	43 (19.5)
2–5 years	92 (22.4)	15 (23.1)	25 (28.7)	7 (18.9)	45 (20.4)
> 5 years	214 (52.2)	32 (49.2)	34 (39.1)	15 (40.5)	133 (60.2)
Severity of acne					
IGA* < 3	190 (46.3)	35 (53.8)	47 (54.0)	14 (37.8)	94 (42.5)
IGA* ≥ 3	220 (53.7)	30 (46.2)	40 (46.0)	23 (62.2)	127 (57.5)

*Investigator global assessment

Women recruited through community and social media advertising had higher acne severity at baseline, with 62.2% (*n* = 23) and 57.5% (*n* = 127), respectively, having an investigator global assessment (IGA) score of 3 or more. These proportions were notably higher compared to participants recruited through primary care (46.2%, *n* = 30) and secondary care (46.0%, *n* = 40). Furthermore, social media advertising yielded a notably different patient profile regarding disease duration. Among women recruited through this method, 60.2% (*n* = 133) reported having acne for over 5 years. This represented a larger proportion compared to other strategies: primary care (49.2%, *n* = 32), secondary care (39.1%, *n* = 34) and community advertising (40.5%, *n* = 15).

Regarding participant ethnicity, primary and secondary care attracted slightly more individuals with White backgrounds (92.3% and 81.6%, respectively) than community (72.9%) and social media advertising (76.9%) approaches.

In absolute terms, social media advertising recruited nearly three times as many women from ethnic minority backgrounds (*N* = 20) compared to the combined primary, secondary and community routes (*N* = 8).

Notably, no significant differences were observed between the recruitment route and participant age, suggesting the various methods were able to reach a similar age distribution.

### Cost implications

From 11 June 2021 to 31 August 2021 (14 months), in-house social media recruitment incurred costs of £6649.30, yielding 209 enrolled participants. This equates to an approximate cost of £31.82 per participant. In contrast, using a mail-out service for participant identification at participant identification centres (PICs) cost £175.26, resulting in 24 enrolled participants at £7.30 per participant.

### Retention

Defined by primary outcome data completeness, participant retention rates varied across recruitment routes at both the 12-week and 24-week follow-up points, with some differences in retention by recruitment method. At the 12-week follow-up visit, which was conducted remotely or face to face, retention was highest among participants recruited through social media advertising (85%), followed closely by primary care recruitment (84%). Community advertising yielded a slightly lower retention rate 79%, while secondary care recruitment showed the lowest retention at this stage, with 73% of participants completing the 12-week measure (Table 3).

**Table 3:** Participant retention by route of recruitment

Timepoint	Route of recruitment			
	Primary care (% retained)	Secondary care (% retained)	Community (% retained)	Social media (% retained)
12 weeks	84	73	79	85
24 weeks	75	68	74	72

By the 24-week endpoint, which only involved a remote questionnaire, retention had declined across all routes, though primary care recruitment shows relatively strong retention (75%), followed by community advertising (74%) and social media (72%). Secondary care recruitment again had the lowest retention rate at this stage (68%).

The variability in retention across recruitment routes highlights the importance of varied recruitment routes to balance initial recruitment with sustained engagement throughout the study. The drop-off in retention over time observed across all routes also signifies the importance of implementing effective retention strategies to keep participants engaged through follow-up.

### Participant perspectives and experiences of social media advertisement and the sign-up process for the SAFA trial

Twelve SAFA trial participants completed an interview (Table 3).

### Targeted advertisements resonated with the audience

Many participants described actively seeking acne-related care online, including through social media platforms. They highlighted the perceived value of the targeted advertisements they saw for the SAFA clinical trial, as the ads were tailored to their specific needs and characteristics, specifically geographic locality, gender, age and condition (Table 4).

**Table 4:** Characteristics of interview participants

Total number	12
Mean age in years (range)	29 (22–36)
Ethnicity	
White	10
Mixed or multiple ethnic groups	1
Prefer not to say	1
Occupation	
Paid employment	10
Self-employed	1
Unemployed	1
Type of trial medication	
Spironolactone	6
Placebo	3
Unsure (not yet unblinded)	3
Route of recruitment	
Facebook/Instagram/Twitter	5
Primary care	2
Secondary care	1
Poster	2
Online chatroom	1
Searched online for trials	1

*I think it was something along the lines of, ‘Are you between…?’ and then a certain age and, ‘…suffering from adult acne?’ I was like, ‘Yes. Me!’* Katy, age 27.

Receiving information about SAFA through social media was generally seen as an acceptable and welcomed way to be made aware and learn about trials and access to new treatment opportunities that they would otherwise might not have known about.

I wish I knew about more trials going on because you’d be able to participate in those. I was searching for a very specific thing, which is how I got to find this trial. Sam, age 25.

A few participants had repeated exposure (two or three viewings) to the social media advert before clicking on it and submitting an expression of interest. They did not report the repeated exposure as bothersome and instead, this seemed to motivate the reader to engage with the advert.

*..it’s come up again. Somebody is saying something here, so maybe I should just try it.* Sam, age 25.

Participants who were not recruited through social media also endorsed this strategy, recognising its potential effectiveness for reaching a wide audience quickly.

*social media is the fastest way with this group to spread information. It really makes sense to try and capitalise on that.* Linda, age 32.

Several participants also mentioned that the advert was forwarded to them, suggesting that showing the advert to a wider audience could also be a snowball sampling strategy to enhance trial recruitment [16].

### Building trust through signals of credibility

Participants highlighted the importance of visually appealing and high-quality graphics in the social media advertisements, as these elements were suggested to help capture their attention and contribute to perceptions of legitimacy. Accounts around making judgements about legitimacy were further supported by recognisable NHS and university logos, as well as linking to further information on the SAFA trial website.

*the NHS logo on it, quite detailed information about the study, contact information so I could then..find a bit more information about it and verify it’s the real thing.* Laura, age 36.

However, some participants expressed a greater degree of scepticism towards social media advertisement than others. A participant who was invited through their GP surgery suggested they would be more likely to trust an invitation that came directly from their healthcare provider.

*I would have definitely been cautious about it [social media advert]…I think it would have really heavily depended on the imagery and where it linked in through.* Kat, age 30.

This suggests that emphasising the credibility of the source may be particularly important in optimising engagement with a social media advert, as well as the use of links to and from trusted websites outside of social media platforms.

### Leveraging existing online communities

Many participants reported being active in international online acne communities, such as Facebook groups and Reddit forums. These spaces offered opportunities to share experiences and learn about various acne treatments. Through their engagement with members from the USA, many participants had already heard of spironolactone, SAFA’s trial intervention, before enrolling but had not been offered it by their GP, despite a few of them directly enquiring about the drug. This familiarity appeared to reduce perceived risks and increase participants’ openness to joining the trial. The exchange of information among people with similar experiences in these communities functioned as a form of organic social proof, building genuine trust and credibility around the trial drug, which may have influenced enrolment decisions. One participant suggested future advertisements could include leveraging the influence of social media figures who could openly discuss their acne journey and share their experience of participating in the trial.

*it’s also looking at social media influencers, if there are any social media influencers that actually have quite bad acne themselves, getting them to do the trial. Then they can then obviously talk about their experience on social, and other people might then want to do it.* Jasmine, age 22.

Developing relationships with influential voices within acne-focused online communities could be a valuable strategy for raising awareness and driving participation.

### Streamlining the onboarding process

Participants recruited through social media advertisements generally experienced a direct and low-effort recruitment process.

*I was worried there might be loads of hoops to jump through and it would end up dragging on for so long that I’d give up! But actually it was really quick and easy to get involved.* Laura, age 36.

The relatively seamless onboarding process, supported by responsive research staff, appeared to play a crucial role in facilitating a positive transition from initial interest to enrolment in the trial.

Those recruited through other pathways, such as secondary care, were unaware of the trial until they were invited by a member of clinical staff.

*I went on to the study was because I went to a dermatologist appointment… I was given an information leaflet to go away with and then the team conducting the study called me and that’s how it all started.* Liz, age 36.

After being invited to participate in the study, participants recruited through all pathways reported that responsive research staff provided a positive experience through to enrolment and were important to their choosing to join the trial.

## Discussion

This study shows that paid, targeted social media recruitment can be an effective and acceptable component of a multi-route strategy for clinical trial enrolment that could reach different segments of a target population. Overall social media accounted for 53.9% of total participants despite a lower conversion rate (0.43%) compared to traditional primary care recruitment (3.16%). Notably, social media recruitment reached a more diverse participant population, recruiting nearly three times as many women from non-White backgrounds compared to combined traditional routes and captured a higher proportion of participants with more severe (IGA ≥ 3) and chronic acne conditions (> 5 years duration, 60.2%). We found social media recruitment to be acceptable to trial participants, who had suggestions regarding what made them click on links and view study advertisements as ‘trustworthy’. While social media reached and recruited most individuals, it required extensive screening efforts, with 540 manual screening calls resulting in 221 recruits. Future studies should explore the impact of self-screening or more autonomous methods to alleviate the screening burden.

Previous research has shown that the success of social media in recruiting participants to clinical trials varies between studies [5]. Our findings suggest that, while social media recruitment may yield lower conversion rates compared to conventional routes, it can rapidly reach large, diverse populations and can effectively support enrolment targets [17]. Our results further develop the evidence base for trial recruitment by providing detailed insights into participants’ perspectives on social media recruitment, highlighting the importance of credibility signals (e.g. NHS branding) and the role of online communities in building trust through social proof. Developing relationships with influential voices within acne-focused online communities could be a valuable strategy for raising awareness and driving participation in future trials.

The findings have important implications for future trial recruitment strategies, particularly considering the NIHR’s emphasis on improving research accessibility and diversity of participants [18]. Supported by responsive research staff, social media recruitment appears to address some traditional barriers to trial participation by providing a direct, low-effort enrolment pathway and reaching historically underserved populations. Additionally, social media recruitment can leverage knowledge exchange and the influence of others (social proof) in existing online health communities and enable repeated exposure to trial information, both of which may help normalise research participation among potential participants.

This study’s strengths include its novel mixed-methods approach, providing both quantitative recruitment metrics and qualitative insights into participant experiences. The integration of multiple recruitment channels allowed for a direct comparison of different strategies’ effectiveness. However, limitations include the small sample size for qualitative interviews (*n* = 12) and potential selection bias, as interviewed participants may have held more positive views about the trial. Furthermore, the sequential implementation of different recruitment strategies (with social media predominantly used from 2020 onwards) may have influenced comparative effectiveness measures. While not a direct limitation of this study, researchers should be mindful of the potential impact of evolving algorithms (instructions that push content and adverts to platform users) on advertising trials on social media platforms. The influence of strategically situated recruiting sites to boost diverse recruitment and the appeal of developed advertisement materials to different segments of a population should also be carefully considered as part of recruitment planning. 

## Conclusions

In conclusion, while social media recruitment demonstrated lower conversion rates than traditional methods, its ability to rapidly reach large, diverse populations and capture participants with more chronic conditions suggests it should be considered an effective and acceptable clinical trial recruitment strategy. Future research should focus on establishing an accurate and standardised way to capture the financial and time demands of different recruitment routes to optimise trial resource allocation.

## Data Availability

The datasets used and/or analysed during the current study are available from the corresponding author on reasonable request.
